# Near telomere-to-telomere genome assemblies of Silkie *Gallus gallus* and Mallard *Anas platyrhynchos* restored the structure of chromosomes and “missing” genes in birds

**DOI:** 10.1186/s40104-024-01141-1

**Published:** 2025-01-20

**Authors:** Qiangsen Zhao, Zhongtao Yin, Zhuocheng Hou

**Affiliations:** Frontiers Science Center for Molecular Design Breeding (MOE), State Key Laboratory of Animal Biotech Breeding, and National Engineering Laboratory for Animal Breeding, College of Animal Science and Technology, China Agricultural University, Beijing, 100193 China

**Keywords:** Avian, Centromere, Missing gene, Telomere-to-telomere genome, 5mC methylation level

## Abstract

**Background:**

Chickens and ducks are vital sources of animal protein for humans. Recent pangenome studies suggest that a single genome is insufficient to represent the genetic information of a species, highlighting the need for more comprehensive genomes. The bird genome has more than tens of microchromosomes, but comparative genomics, annotations, and the discovery of variations are hindered by inadequate telomere-to-telomere level assemblies. We aim to complete the chicken and duck genomes, recover missing genes, and reveal common and unique chromosomal features between birds.

**Results:**

The near telomere-to-telomere genomes of Silkie *Gallus gallus* and Mallard *Anas platyrhynchos* were successfully assembled via multiple high-coverage complementary technologies, with quality values of 36.65 and 44.17 for Silkie and Mallard, respectively; and BUSCO scores of 96.55% and 96.97% for Silkie and Mallard, respectively; the mapping rates reached over 99.52% for both assembled genomes, these evaluation results ensured high completeness and accuracy. We successfully annotated 20,253 and 19,621 protein-coding genes for Silkie and Mallard, respectively, and assembled gap-free sex chromosomes in Mallard for the first time. Comparative analysis revealed that microchromosomes differ from macrochromosomes in terms of GC content, repetitive sequence abundance, gene density, and levels of 5mC methylation. Different types of arrangements of centromeric repeat sequence centromeres exist in both Silkie and the Mallard genomes, with Mallard centromeres being invaded by CR1. The highly heterochromatic W chromosome, which serves as a refuge for ERVs, contains disproportionately long ERVs. Both Silkie and the Mallard genomes presented relatively high 5mC methylation levels on sex chromosomes and microchromosomes, and the telomeres and centromeres presented significantly higher 5mC methylation levels than the whole genome. Finally, we recovered 325 missing genes via our new genomes and annotated *TNFA* in Mallard for the first time, revealing conserved protein structures and tissue-specific expression.

**Conclusions:**

The near telomere-to-telomere assemblies in Mallard and Silkie, with the first gap-free sex chromosomes in ducks, significantly enhanced our understanding of genetic structures in birds, specifically highlighting the distinctive chromosome features between the chicken and duck genomes. This foundational work also provides a series of newly identified missing genes for further investigation.

**Supplementary Information:**

The online version contains supplementary material available at 10.1186/s40104-024-01141-1.

## Background

Chickens and ducks are the two most farmed poultry, providing a significant amount of animal protein and occupying an important position in human society. However, previous studies have suggested that a significant number of protein-coding genes are missing in avian genomes compared with mammalian and amphibian genomes [1, 2]. Sustained efforts are being made to recover these "missing" genes, which may have been overlooked in incomplete genomes [3–5], especially complex regions such as centromeres and telomeres, which can be resolved through telomere-to-telomere (T2T) genomes. In recent years, an increasing number of pangenome studies [6, 7] have shown that one single genome is not sufficient to represent all the genetic information of a species. This suggests that a single reference genome impedes the discovery of functional genes, and more complete genomes of different breeds are needed to characterize the genomes of avian species collectively. Comparative genomics can help us identify similarities and differences between species. Microchromosomes exist in bird genomes, but the properties of microchromosomes, such as centromere composition and 5-methylcytosine (5mC) methylation levels relative to macrochromosomes, are still unclear. Centromeres are repeat-rich heterochromatic regions critical for faithful chromosome segregation during cell division [8]. The sequence and structure of centromeric regions are highly diverse among different species. Compared with conventional genome assemblies, T2T genomes have significant advantages, primarily reflected in the completeness and accuracy of the genome assembly, the discovery of functional genes, and the detection of structural variants. Therefore, we aimed to enhance the genomes of chickens and ducks to the T2T level and employ comparative genomics to study the differences and commonalities between chickens and ducks in terms of chromosome types, centromeres, transposable elements, and 5mC methylation. Furthermore, utilizing the T2T genomes of chickens, ducks and other published avian genomes together, we can investigate extensively and recover missing genes that were previously thought to be in avian species.

## Methods

### Sample collection and sequencing

To achieve T2T genome assembly, we have added new sequencing data in addition to the existing data from both Silkie and Mallard from our previous work [9, 10]. Fresh blood from the same individual was used for high-fidelity (HiFi) sequencing and Oxford Nanopore Technology (ONT) sequencing of the Mallard. Information related to the sequencing summary of the Mallard is shown in Table S1 and Fig. S1. Fresh blood from the same individual was used for nanopore sequencing of Silkie. Information related to the sequencing summary of Silkie is shown in Table S2 and Fig. S1. The DNA from the same Silkie used to generate the ONT sequencing libraries was the same as that used for the Mallard.

To construct sequencing libraries for Pacific Biosciences (PacBio) HiFi sequencing, more than 20 µg of sheared DNA was subjected to size selection via the Blue Pippin system, and ~ 15 kb Sequel SMRT bell libraries were prepared according to the protocol provided by the PacBio company. Four SMRT cells were run on a PacBio RSII system via P6‒C4 chemistry. Genomic DNA for ONT read sequencing was isolated from the blood. DNA was extracted via the phenol:chloroform:isoamyl alcohol (25:24:1) method from the Tris + SDS (sodium dodecyl sulfate) + EDTA + NaCl lysing reagent-treated tissues without a purification step to ensure a sustained length of genomic DNA. The sequencing libraries were processed via a Ligation Sequencing 1D Kit (SQK-LSK109, Oxford Nanopore Technologies, UK) according to the manufacturer’s instructions. Four DNA libraries were constructed and sequenced on the PromethION platform (Oxford Nanopore Technologies, UK). Guppy (v5.0) was used for base calling and output to FASTQ files.

### Genome assembly and assessment

After trying a variety of strategies for assembly, we integrated a method suitable for assembling, correcting, and gap-filling bird microchromosomes. The assembly pipelines are shown in Fig. S2 and S3. For both CAU_Silkie_2.0 and CAU_Wild_2.0, we integrated multiple data sources and used a manual assembly pipeline based on HiFi phased assembly to merge contigs from multiple data sources and methods based on genome collinearity.

Specifically speaking, for CAU_Silkie_2.0, PacBio subreads were filtered and corrected with the circular consensus sequencing (CCS) pipeline v6.0.0 (https://github.com/PacificBiosciences/ccs). Then, adapters of HiFi reads were filtered by HiFiAdapterFilt (v2.0.1) [11], adapters of the ONT reads were trimmed via PoreChop (v0.2.4) [12]; the preprocessing of Hi-C reads was completed via fastp (v0.20.1) [13]. The HiFi reads, ONT reads longer than 50 kb in length and Hi-C reads were subjected to hifiasm (v0.19.6) [14] for double-graph phased assembly. HiFiasm phased contigs were used to phase ONT reads longer than 30 kb by mapping with minimap2 (v2.26) [15]. All ONT reads longer than 30 kb and phased reads were subsequently subjected to NextDenovo (v2.5.2) [16] and CANU (v2.2) [17]. The quality of Hi-C reads was controlled by HiC-Pro (v3.0) [18], and only valid pairs were used for subsequent analysis. The primary HiFiasm contigs were used for scaffolding with Hi-C reads via YAHS (v1.2a.2) [19]. Hi-C reads were mapped with Chromap (v0.2.5-r473) [20]. Contigs from ONT reads were rescued by picking up contigs without mapping with any scaffolds. Scaffolds were manually curated by JuiceBox (v1.11.08) [21] with a Hi-C interaction signal and collinearity with CAU_Silkie_1.0. Gap filling is completed step by step with various versions of contigs and various types of reads. First, chromosomal structures are corrected by comparing different versions of contigs, Hi-C signals, and collinearity with CAU_Silkie_1.0. LINKVIEW2 (https://yangjianshun.github.io/LINKVIEW2/) and scripts from GitHub (https://github.com/ZhouQiLab/DuckGenome/tree/master/anchoring_chr, referred to as anchor scripts) were utilized to manually inspect and integrate sequences into chromosomes or link chromosomes together. Reads are subsequently utilized to patch the remaining gaps via TGS-Gapcloser (v1.2.0) [22]. The remaining gaps were closed through a combination of long reads or contigs that span both ends of the gap. This process was facilitated by scripts from GitHub (https://github.com/zhangleiworld/gapfill_by_reads), DEGAP [23] and anchor scripts, all under manual inspection. After this, duplications were purged with purge_dups (purged with HiFi reads, manually checked cutoffs, v1.2.6) [24]. Contaminations were selected by Krakenuniq (v1.04) [25] leveraging reference databases comprising human, vector, and microbial sequences. The scaffolds were subsequently polished for 2 rounds via HiFi reads with NextPolish (v1.4.1) [26]. Mitochondrial assembly was performed with MitoHiFi (-o 2, v3.0.0) [27].

For CAU_Wild_2.0, a similar pipeline was used. Additionally, CLR reads were downsampled to include only those longer than 17 kb via Filtlong (–min_length 17,000, v0.2.1, https://github.com/rrwick/Filtlong) and assembled by NextDenovo. Illumina reads were assembled via megahit (v1.2.9) [28]. RunBNG (v1.03) [29] was employed to further scaffold the scaffolds. This was achieved by integrating hybrid assembly with Bionano optical maps. Contigs from CLR and ONT were rescued by picking up contigs without mapping with any scaffolds. HiFi reads and WGS reads were used to polish the genome for 2 rounds with NextPolish2 (v0.2.0) [30] and Pilon (v1.24–0) [31], respectively.

Benchmarking Universal Single-Copy Orthologs (BUSCO) (aves_odb10, *n* = 8,338, v5.0.0) [32] was used to assess the completeness and accuracy of the assembled genome. To test the consistency between the raw data and the assembly, we aligned all the reads back to the genomes. For CAU_Silkie_2.0, we calculated the quality value (QV) from merqury (v1.3) [33] with HiFi reads, while for CAU_Wild_2.0, the QV was calculated from Illumina reads.

### Centromere and telomere identification

We searched for the presence of telomere repeats (TTAGGG)n via quarTeT (v1.03) [34]. The ChIP-seq data of CENPA were aligned with the BWA-MEM algorithm with options “-k 50 -c 1000000”. The alignment duplications were marked with sambamba (v0.6.3) [35] and filtered with samtools (view -q 30 -F 2308, v1.15.1). We counted the reads with BEDTools genomecov (v2.29.2) [36]. To annotate the putative centromeres of CAU_Wild_2.0, we searched the genome with the reported 190-bp duck centromeric repeats [37] using TRFinder (2 5 7 80 10 50 2000, v4.09) [38] and SRF [39] followed by manual curation. Similarity heatmaps were generated via StainedGlass (v0.6) [40].

### Genome structure prediction and annotation

We mapped the RNA-seq data (Table S3) against the genome assembly with HISAT2 (v2.1.0) [41]. The transcripts were assembled via StringTie (v2.0) [42]. TransDecoder (v5.5.0, https://github.com/TransDecoder/TransDecoder) was used to predict protein-coding regions of the assembled transcripts. Gene models were annotated via the EVidenceModeler (EVM) genome annotation pipeline (v2.31.8) [43], which integrates both ab initio gene predictions generated by Braker3 (v2.1.6) [44] and Helixer (online server) [45], protein-coding regions of the genome-guide assembly of transcripts in the genome, and homology evidence, including protein sequences in the SwissProt database, via exonerate (v2.4.0) (https://github.com/nathanweeks/exonerate). The gene models were further refined twice via PASA (v2.4.1) [46]. To assess the completeness and accuracy of the annotations, we computed BUSCO scores for the annotations using compleasm (v0.2.2) [47].

### Identification of noncoding RNA genes

Noncoding RNA species, including microRNA (miRNA), transfer RNA (tRNA), ribosomal RNA (rRNA) and small nuclear RNA (snRNA), were annotated via several methods. tRNAs were predicted via tRNAscan-SE (v1.3.1) [48] with default parameters before repeat masking. miRNAs and snRNAs were annotated by scanning Rfam (v14.0) [49] against the genome and passing the results into Infernal (v1.1.3) [50] with default parameters. The results are shown in Table S4.

### Annotation of repeats and transposable elements

Repeats were analyzed via a method that combines de novo structure analyses and homology comparisons. First, RepeatModeler (-LTRStruct, 2.0.2a) [51] was employed to construct the repeat element library. The repeat regions were then annotated via RepeatMasker (v4.1.2-p1) [52] via the repeat library generated from combining de novo prediction, the reference library (Dfam and Repbase) and the avian repeat library [53]. Repetitive elements accounted for 15%–17% of the genome, most of which were long interspersed nuclear elements (Table S5). ClassifyTE [54] was used to classify unclassified transposable elements. TRASH (v1.2) [55] was used to identify and extract tandem repeats in genome sequences and investigate their higher-order structures.

### DNA methylome analysis

DNA 5mC methylation was called with Nanopolish (-q cpg, v0.13.2) [56] by using the Hidden Markov Model. ONT fast5 files were used as the input files. The methylation frequency was calculated as the number of reads on methylated cytosine divided by the total number of reads covering each cytosine site in the reference.

### Strategy to identify missing genes

We used the proteins of the assembled genomes to find sequences homologous to any of the 571 proteins of genes previously thought to be missing in the bird genomes, of which 274 were thought to be missing from all avian genomes [1–3, 5, 57]. The human protein sequences of the corresponding missing genes were used as query sequences to search for homologs in the newly assembled Silkie and Mallard genomes via the reciprocal best-hit algorithm with Mmseqs2 (Release 15-6f452) [58]. We manually checked each matched candidate sequence based on the list of missing genes to distinguish synonyms, paralogs, and alignment errors. We used the AlphaFold 3 server [59] to predict the protein conformation of *TNFA* with seed 12346. Finally, JCVI (v1.3.9) [60] was used to plot gene collinearity.

## Results

### Near telomere-to-telomere genome assembly and completeness evaluation

To achieve complete assembly of the genomes of Silkie and Mallard, we adopted multiple high-coverage complementary technologies. The CAU_Silkie_2.0 genome was assembled by incorporating ONT and PacBio HiFi long-read sequences as well as sequences from high-throughput chromatin conformation capture (Hi-C) technologies (~ 39X HiFi, ~ 245X ONT and ~ 193X Hi-C, Table S2), and the N50 of ONT reads reached 33 kb (Fig. S1). While for CAU_Wild_2.0, in addition to HiFi and ONT, Hi-C sequences also include PacBio Continuous Long read (CLR) sequences, BioNano Optical Maps (BOMs), and Illumina sequences (~ 36X HiFi, ~ 207X ONT, ~ 88X BOM, ~ 93X CLR, ~ 116X Hi-C and 121X Illumina, Table S1), and the N50 of ONT reads reaches 32.6 kb (Fig. S1). Multiple complementary high-depth sequencing datasets can effectively ensure the continuity, completeness, and accuracy of the assembly. For both CAU_Silkie_2.0 and CAU_Wild_2.0, we integrated multiple data sources and used a manual assembly pipeline based on HiFi phased contigs by overlapping contigs (Table S6 and S7) from multiple data sources and assembly software (Fig. S2 and S3).

**Fig. 1:**
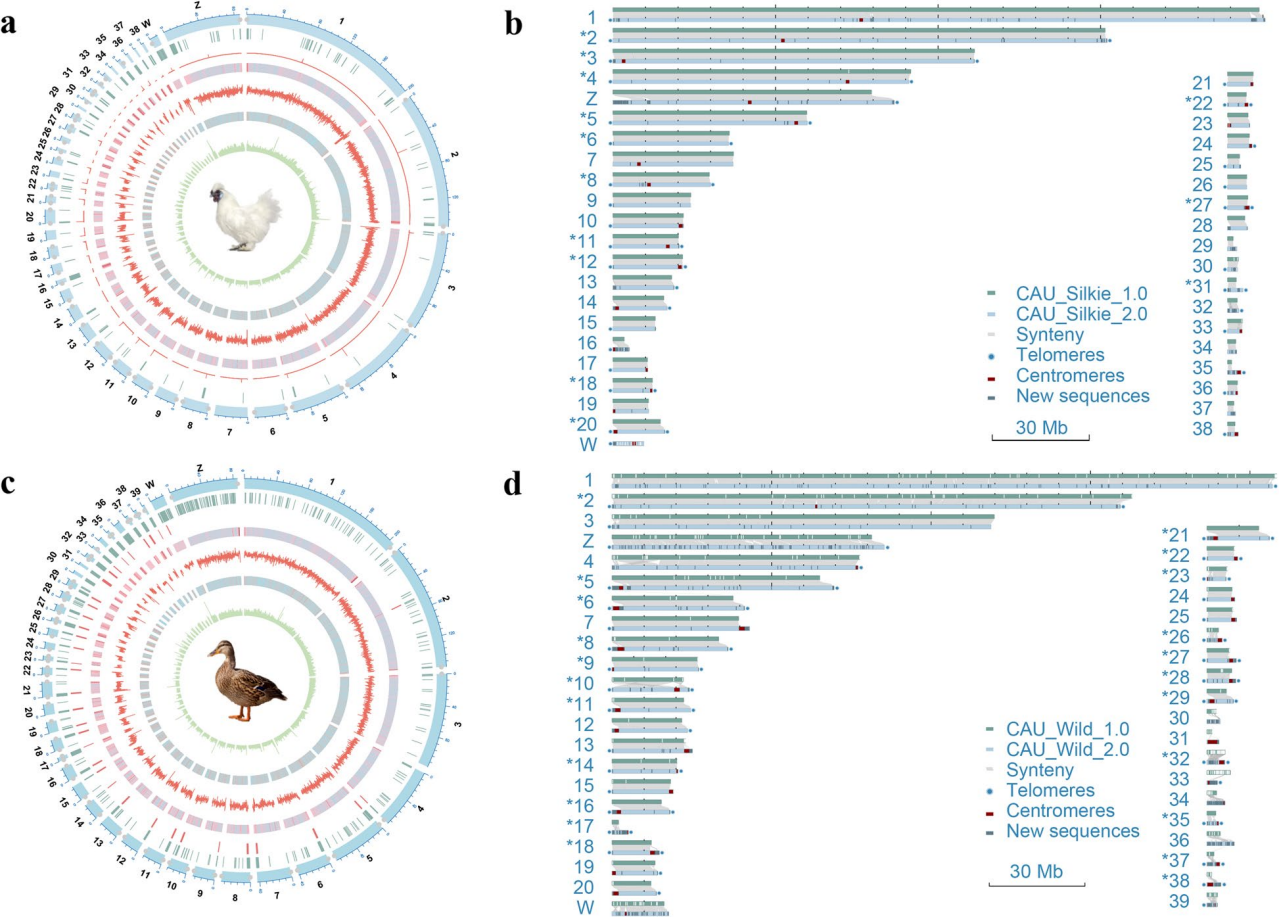
Genome landscape and comparative genome collinearity plot. **a** Circle plot of CAU_Silkie_2.0. **b** Collinarity plot of CAU_Silkie_2.0 vs. CAU_Silkie_1.0. **c** Circle plot of CAU_Wild_2.0. **d** Collinarity plot of CAU_Wild_2.0 vs. CAU_Wild_1.0. From outer to inner: chromosome length (unit: Mb), telomeres (gray dots); new sequence compared with CAU_Silkie_1.0 (vertical lines); peak signal of CENPA (pink lines) for CAU_Silkie_2.0; locations of centromeric repeats (pink vertical lines) for CAU_Wild_2.0; GC content (red color means higher GC percentage; light blue color means lower GC percentage); 5mC methylation level; gene density (red color means higher gene density; light blue color means lower gene density); number of TEs; the appearance. The asterisks before the chromosome numbers indicate that the chromosomes are T2T gap-free assemblies

The final genome size of CAU_Silkie_2.0 is 1.09 Gb, with a scaffold N50 size of 90.91 Mb (Table S8). A total of 1.08 Gb (99.03%) of genome sequence was further assigned to 40 chromosomes with only 12 gaps, including 36 gap-free chromosomes and 16 T2T chromosomes (Fig. 1a and b, Table S9). Compared with CAU_Silkie_1.0, the W chromosome was rescued (Fig. S4). By comparing CAU_Silkie_2.0 with CAU_Silkie_1.0, we found that the telomere and subtelomere regions were also rescued on Chr1, ChrZ, Chr31 and Chr35 (Fig. 1b, Table S9). The Hi-C interaction signals from the genome indicate the absence of large-scale structural errors (Fig. S5). Furthermore, the near T2T assembly contained a total of 33 Mb of new sequences ranging from 7 kb to 6.59 Mb per chromosome, which was absent in CAU_Silkie_1.0 chromosomes with extremely high GC contents or extremely high AT contents (Fig. 1b, Fig. S6b, Table S10).

The final genome size of CAU_Wild_2.0 is 1.22 Gb, with a scaffold N50 size of 76.95 Mb (Table S8), becoming the best quality duck genome. A total of 1.21 Gb (99.06%) of genome sequences were further assigned to 41 chromosomes with only 3 gaps, including 39 chromosomes that are gap-free and 23 chromosomes that are T2T (Table S11), a significant decrease in gap number (3% vs. 318, 99%) compared with CAU_Wild_1.0, inversions on Chr4, Chr10, and ChrW were corrected in CAU_Wild_2.0, and the centromeres of 36 chromosomes were identified in CAU_Wild_2.0 (Fig. 1c and d, Table S12). The Hi-C interaction signals from the genome indicate the absence of large-scale structural errors in the assembly (Fig. S7). Furthermore, the near T2T assembly contained a total of 72 Mb of new sequences from 219 kb to 4.26 Mb, which were absent in CAU_Wild_1.0 chromosomes with extremely high GC contents or extremely high AT contents (Fig. 1d, Fig. S6a, Table S10).

To further evaluate the completeness of chromosome assembly, we searched for the presence of telomere repeats (TTAGGG)n and centromeres within CAU_Wild_2.0, CAU_Wild_1.0, and SKLA1.0 [61] for comparison, SKLA1.0 is a chromosome-scale Pekin duck (*Anas platyrhynchos*) assembly generated recently. We found that the telomere repeats were present at the ends of 36 chromosomes in CAU_Wild_2.0 (Fig. 1c and d), with an average length of 10.90 kb (a total of 446.88 kb, Table S11), but few telomere repeats were observed within CAU_Wild_1.0 and SKLA1.0 (Fig. S8 and S9). The centromere sequences were predicted on 36 chromosomes of CAU_Wild_2.0 (Fig. 1c and d; Table S12), but few centromere sequences have been observed within CAU_Wild_1.0 and SKLA1.0 (Fig. S8 and S9). We also found that telomere repeats were present at the ends of 36 chromosomes in CAU_Silkie_2.0, with an average length of 8.44 kb (a total of 337.77 kb, Table S9, Fig. 1a and b). Functional centromeres can be determined from ChIP-seq data of centromere protein A (CENPA), which is available for chickens. We downloaded related data [62] from chickens and detected functional centromeres across the entire genome. There are 24 chromosomes in CAU_Silkie_2.0 with peaks where functional centromeres are located (Fig. 1a and b; Table S13).

We assessed the genome from BUSCO, QV, and read alignment rates. BUSCO scores revealed that CAU_Silkie_2.0 (96.55%) and CAU_Wild_2.0 (96.97%) achieved superior assembly quality (Table S14). For CAU_Silkie_2.0, the quality value reached 36.65, leading to a base accuracy of 99.978%. The mapping rates of HiFi and ONT reads achieved 99.52% and 99.63%, respectively, also mapping rates of the reads from GGswu (Huxu chicken, *Gallus gallus*) achieved 99.92% (HiFi) and 99.40% (ONT). And for CAU_Wild_2.0, the QV reached 44.17, leading to a base accuracy of 99.99627%; the mapping rates of ONT, HiFi and Illumina reads achieved 99.75%, 99.60% and 99.89%, respectively, and mapping rates of the reads from SKLA1.0 (Pekin duck, *Anas platyrhynchos*) achieved 99.74% (Illumina) and 99.51% (ONT). The aforementioned indicators show that the two assemblies ranked in the first tier among bird genomes.

### Annotation of repetitive elements, noncoding RNAs, and protein-coding genes

Repetitive element annotation revealed that 17.62% (21.8 Mb) and 15.17% (16.6 Mb) of the CAU_Wild_2.0 and CAU_Silkie_2.0 elements are composed of repetitive elements, respectively, and long interspersed nuclear elements (LINEs) constitute the largest class of transposable elements annotated in both CAU_Wild_2.0 and CAU_Silkie_2.0; other predominant repetitive elements are summarized in Table S5. Noncoding RNA was also detected, accounting for 0.01% of both CAU_Wild_2.0 and CAU_Silkie_2.0 (Table S4), 244 miRNA, 521 tRNA, 264 rRNA and 297 snRNA were annotated in CAU_Wild_2.0; 255 miRNA, 318 tRNA, 60 rRNA and 296 snRNA were annotated in CAU_Silkie_2.0 respectively. The protein-coding genes were subsequently annotated via a combination of ab initio, homology-based, and transcript evidence prediction approaches. For transcript evidence, 42 tissues and 16 tissues (Table S3) were used for CAU_Silkie_2.0 and CAU_Wild_2.0, respectively, and a total of 20,264 and 19,621 genes were successfully identified from CAU_Silkie_2.0 and CAU_Wild_2.0, respectively. After gene structural annotation, InterPro, PANZER2, EggNOG, SwissProt, and NR were employed for gene functional annotations, and 18,697 (92.27%) and 18,574 (94.66%) genes were mapped to at least 1 database for CAU_Silkie_2.0 and CAU_Wild_2.0, respectively (Table S15). Evaluation of completeness and accuracy of annotation showed high-quality results for both chicken (94.78%) and duck annotations (96.03%, Table S16).

Notably, gap-free sex chromosomes (ChrW and ChrZ) were assembled for the first time in Mallard. Good gene collinearity was identified with a greater number of new genes (Fig. S10). There were 864 and 182 protein-coding genes for ChrZ and ChrW, respectively; among them, 805 and 149 genes with functional annotations for ChrZ and ChrW, respectively, and 96 new genes with complete open reading frames (ORFs) were compared with CAU_Wild_1.0 in total. (Table S17 and S18, Fig. S10).

### Differences between macro- and microchromosomes and diverse centromere types

**Fig. 2:**
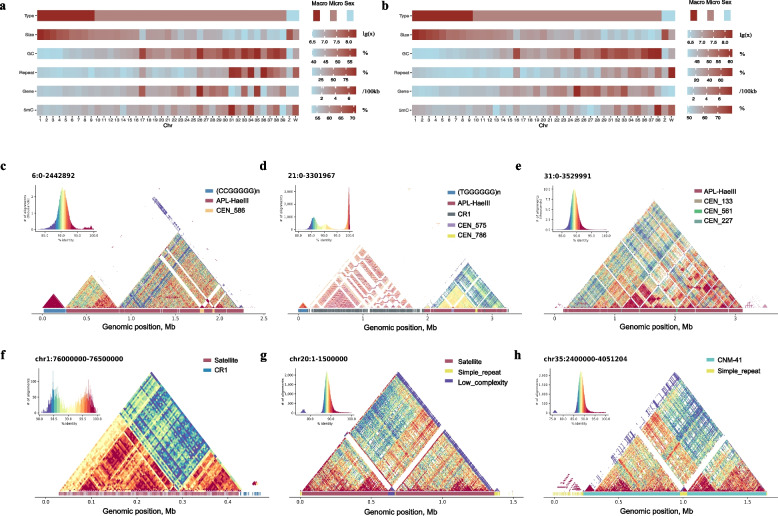
The various attributes of chromosomes and the classification of centromeres. **a** Attribute landscape of CAU_Wild_2.0. **b** Attribute landscape of CAU_Silkie_2.0. From top to bottom: chromosome type, chromosome size (in log scale), GC content, repeat sequence content, novel sequence content compared with CAU_Wild_1.0, average number of genes per 100 kb window, and average 5mC methylation level. **c–h** Heatmaps illustrate the sequence similarity of centromeric regions in (**c**) Chr6, (**d**) Chr21, (**e**) Chr31 chromosomes of the Mallard genome; and (**f**) Chr1, (**g**) Chr20, (**h**) Chr35 of Silkie genome along with the distribution of their repetitive sequence types

A comparison of the newly assembled near T2T avian genomes of CAU_Wild_2.0 and CAU_Silkie_2.0 with their previous versions revealed that the majority of centromeric and telomeric sequences (59/82, 36/42; 48/80, 24/40; Tables S9, S11, S12, and S13) were identified. By utilizing near T2T genomes, we also identified differences in avian genomes between macrochromosomes and microchromosomes, including differences in GC content, repeat sequence content, gene density, and the 5mC methylation level (Fig. 2a and b). In both Silkie and Mallard, the microchromosomes tended to present the following characteristics: higher GC content, a greater proportion of repetitive sequences, higher gene density, and a higher level of 5mC methylation (Fig. 2a and b) than macrochromosomes.

We focused on newly assembled sequences, i.e., centromeres, and our comparative analysis of centromere repeat sequence structures in Silkie and Mallard revealed that their genome centromere structures can essentially be categorized into three types. In Mallard, the inherent type APL-HaeIII is present in the centromeres of almost all chromosomes (Fig. 2c–e), and the centromeres of Chr5 and Chr21 have been invaded by chicken repeat 1 (CR1) transposable elements (Fig. 2d, Fig. S11). In the chicken genome, centromeric regions are composed primarily of satellite sequences, CNM-41 [63] sequences, and simple repeats. The dominant portions of the repetitive sequences transition from satellite sequences in macrochromosomes to CNM-41 sequences in microchromosomes. In addition to centromeres with tandem repeat sequences, we also obtained centromeres from Chr5, ChrZ, and Chr27 without tandem repeat sequences in the chicken genome [62].

### Highly heterochromatic W chromosomes serve as refuges for ERV accumulation

**Fig. 3:**
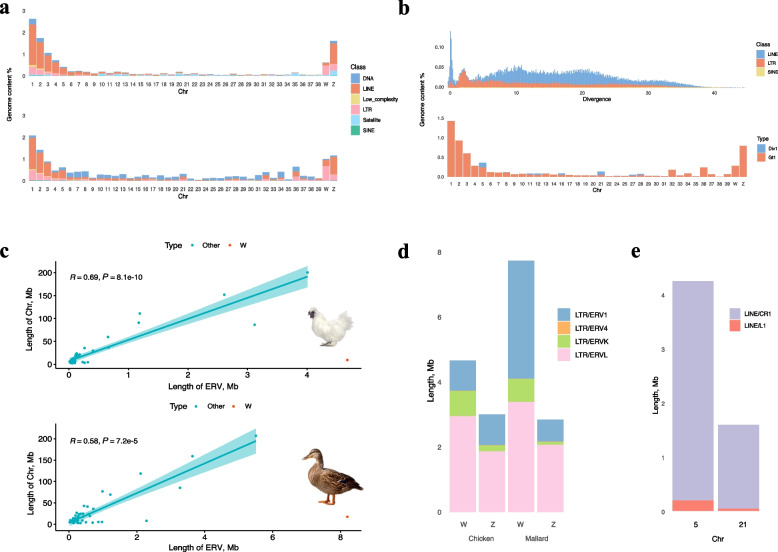
The characteristics of TEs in the Silkie and the Mallards. **a** Abundance distribution of TEs across the whole genome. The top panel is CAU_Silkie_2.0, and the lower panel is CAU_Wild_2.0. **b** Active LINEs and their distribution on chromosomes. Div1 represents a divergence of less than or equal to 1%, and Gt1 represents a divergence greater than 1%. **c** Disproportionate content of LTRs on the W chromosome. **d** Subtypes of LTRs on the W chromosome and **e** subtypes of active LINEs

The relatively large genome size is also accompanied by a relatively high content of repetitive sequences; the Mallard genome has approximately 2% more repetitive sequences than the chicken genome does (Table S5). Upon categorization of the newly identified, unclassified repetitive sequences before, we discerned that the Mallard genome encompasses 5.29% of the DNA transposons (higher than the chicken genome by 4.26%), which are relatively evenly dispersed across all chromosomes, in contrast to the chicken genome, where they are predominantly located on the macrochromosomes (Fig. 3a). Moreover, active transposable elements of the LINE, which primarily target the centromeres of Chr5 and Chr21 for transposition, were identified as mentioned above (Fig. 3b). For both the chicken and the Mallard genomes, W chromosomes contained disproportionately high amounts of endogenous retroviruses (ERVs), with lengths exceeding 4.5 Mb and 8 Mb (47.87% and 45.45%, respectively) (Fig. 3c). Additionally, the type of LINE sequence activated in the Mallard was identified as CR1 (Fig. 3d). Upon systematic verification, we discovered that only *ZDHHC20* and *RRP9* were inserted by active CR1 elements, which may impact the function of those genes. Comparative analysis revealed that the primary ERV type in the Silkie W chromosome is ERVL, whereas in the Mallard chromosome, it is mainly ERV1 and ERVL (Fig. 3d); also, we found that the subtype of active LINEs in Mallard genome is CR1, mainly located in Chr5 and Chr21 (Fig. 3e).

### Relatively high methylation levels of sex chromosomes, microchromosomes, centromeres, and telomeres

**Fig. 4:**
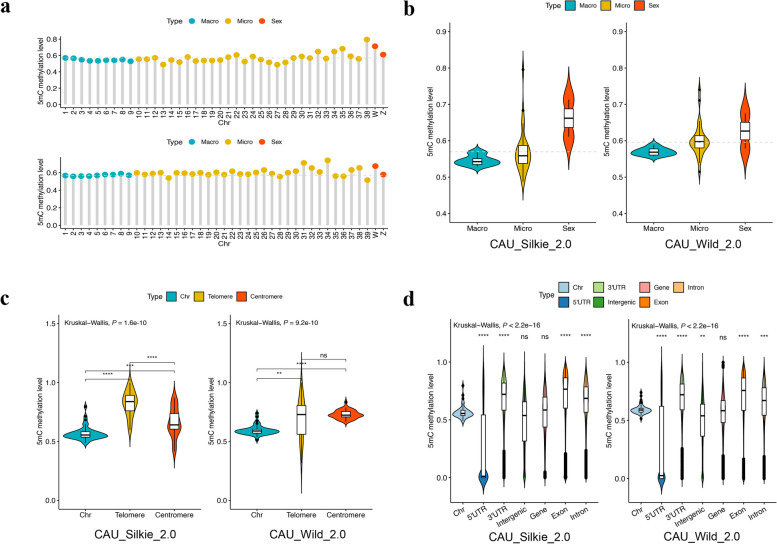
5mC methylation landscape of Silkie and Mallard genomes. **a** Average 5mC methylation levels of chromosomes from Silkie and Mallard. The upper panel is CAU_Silkie_2.0, and the lower panel is CAU_Wild_2.0. The light gray dashed line represents the average level of the genome. **b** Comparison of 5mC methylation levels between categories of chromosomes. Comparisons between designated groups were conducted via the Wilcoxon test. Comparisons among multiple groups were performed via the Kruskal-Wallis test. *: *P* < 0.05, **: *P* < 0.01, ns: *P* > 0.05. **c** and **d **The light gray dashed line represents the average level of the genome. Comparison of 5mC methylation levels between (**c**) chromosomes and other chromosome components, (**d**) chromosomes and other gene context components. Comparisons between designated groups were conducted via the Wilcoxon test. Comparisons among multiple groups were performed via the Kruskal-Wallis test. **: *P* < 0.01, ***: *P* < 0.001, ****: *P* < 0.0001, ns: *P* > 0.05

From the perspective of average 5mC methylation levels across whole-genome chromosomes, the average 5mC methylation level of the duck genome was slightly greater than that of the chicken genome (0.5919 vs. 0.5698, 4.58% greater, Fig. 4a). When we focused on the differences between chromosome types, we observed that, in both Silkie and the Mallard genomes, the methylation levels of the sex chromosomes and microchromosomes were greater than those of the macrochromosomes (Silkie: 21.16% and 4.44%, respectively; the Mallard: 9.93% and 5.52%, respectively; Fig. 4b). For the newly assembled telomeres and centromeres, we also compared their methylation levels with those of the whole genomes of Silkie and Mallard. As anticipated, these gene-poor deserts, which are rich in repetitive sequences, presented significantly higher methylation levels than did the whole genome (Fig. 4c).

For the gene context region, we found that only the 5′ untranslated region (5′ UTR) presented significantly low methylation relative to the average methylation level of chromosomes (Fig. 4d). The 5′ UTR is a regulatory region of DNA situated at the 5′ end of all protein-coding genes that are transcribed into mRNA but not translated into protein. This region contains various regulatory elements and plays a major role in controlling translation initiation [64].

### Recovery of “missing genes” from the newly assembled genomes

**Fig. 5:**
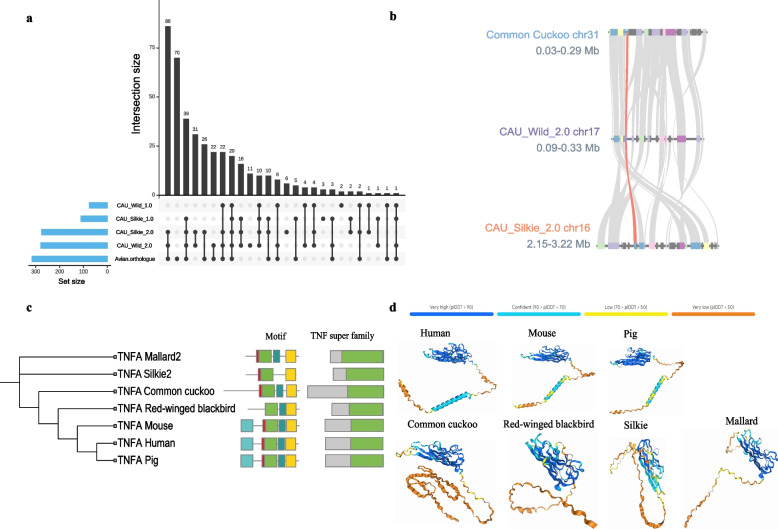
The missing genes identified in the new Silkie and Mallard genomes and the confirmation of the *TNFA* gene. **a** The presence of 571 genes previously reported as missing in birds has been investigated in the chicken and the Mallard genomes. Among them, *TNFA* was identified for the first time in ducks. **b** Gene collinearity of the *TNFA* gene in Silkie, Mallard, and Cuckoo. The orange link represents *TNFA*. **c** Evolutionary relationships of TNFAs across species, motifs, and conserved structural domains of proteins. **d** Prediction of the three-dimensional conformation of the protein encoded by *TNFA*. Different colors represent the predicted confidence levels

By utilizing the new genome along with genomes from RefSeq, we revisited the list of missing genes. A total of 325 (56.9%) missing genes were identified from the new genomes (Fig. 5a, Table S19). By searching for missing genes from a broader perspective (all avian genomes from RefSeq), we found that 315 genes (55.1%) could be found in the avian orthologous gene database of RefSeq (Table S19), and when combined with our results, a total of 401 (70.2%) genes could be recovered (Table S18). By observing the distribution of the missing genes recovered in this study on the chromosomes, we found that the missing genes are concentrated mainly in the centromeres, telomeres, acrocentric chromosomes, and microchromosomes, which are difficult to assemble (Chr12, Chr14, Chr16, Chr29-38 of CAU_Silkie_2.0, Chr2, ChrZ, Chr17, Chr30, Chr33, Chr35, Chr37-39 of CAU_Wild_2.0, Fig. S12 and S13), suggesting that the reason for the absence of genes could not be found previously because of the difficulty in assembling certain highly heterochromatic microchromosomes completely, resulting in these adjacent gene blocks being missing in a block manner.

Tumor necrosis factor alpha (*TNFA)* is a pleiotropic cytokine that plays a significant regulatory role in avian energy metabolism, insulin sensitivity, appetite, and disease pathogenesis [65–67]. Although the *TNFA* gene in chicken genomes has been annotated manually on Chr16 [9], the *TNFA* gene in ducks has still not been annotated from published genomes. Here, *TNFA* was annotated from both our newly assembled Silikie and Mallard genomes, as well as from previously published genomes CAU_Silkie_1.0 (Fig. S19). To validate the accuracy of our assembly and annotation of *TNFA*, we analyzed the gene collinearity between the annotated *TNFA* gene in the cuckoo and the *TNFA* gene identified in this study. The strong gene collinearity among them confirms the accuracy of the *TNFA* in this study and the precision of our assembly and annotation process (Fig. 5b). In addition, we confirmed the identification of *TNFAs* via phylogenetic trees, motif analysis, and analysis of conserved protein domains. These findings indicate that the protein sequence of *TNFA* is conserved with that found in mammals (Fig. 5c). To understand the expression pattern of *TNFA* in ducks, we quantified its expression across 19 tissues in ducks (Table S20). The results revealed that *TNFA* was most highly expressed in the brain and spleen, not expressed in the liver, and expressed in all other examined tissues, corresponding with its role as a pleiotropic cytokine in biological functions (Fig. S14). Additionally, we predicted the protein conformation of *TNFA* and found that the proteins encoded by *TNFA* in different species exhibited similar conformations within conserved structural domains with the highest confidence ratings (blue and light blue, Fig. 5d). These findings suggest that the functions of the proteins encoded by *TNFA* are highly conserved across species.

For several genes that have not been previously annotated in chickens and ducks but have garnered significant research interest, we have, for the first time, successfully assembled and annotated these genes in both Silkie and the Mallard. For example, *BAX* encodes proteins that undergo a conformation change that causes translocation to the mitochondrial membrane, leading to the release of cytochrome c, which then triggers apoptosis under stress conditions [68]; *CFP* encodes a plasma glycoprotein that positively regulates the alternative complement pathway of the innate immune system [69]; and *GAPDHS* encodes a protein that belongs to the glyceraldehyde-3-phosphate dehydrogenase family of enzymes, which may play an important role in regulating the switch between different energy-producing pathways during spermiogenesis and is required for sperm motility and male fertility [70] (Table S19).

## Discussion

We successfully assembled and annotated near T2T genomes for Silkie and Mallard by using multiple high-coverage complementary technologies, including a gap-free pair of ZW sex chromosomes in ducks, a milestone not previously achieved in avian genomic research. A review of the latest studies, such as SKLA1.0 (Fig. S9) and the nearly complete chicken genome GGswu [71], revealed that no research has compiled complete ZW chromosomes. In the Silkie genome, ChrZ was assembled without gaps, whereas the W chromosome still exhibited some gaps, potentially resolvable with longer ONT ultralong reads (N50 greater than 100 kb). This assembly situation parallels that of the W chromosome in GGswu chickens [71].

Furthermore, we utilized the centromere structures of Silkie and Mallard for comparative analysis for the first time, revealing novel centromeric repeat sequences (Fig. 2c–e). Notably, CR1 has infiltrated the centromeric region of the Mallard genome alongside the previously identified APL-HaeIII [37]. In the Silkie genome, there is a transition from satellite sequences in macrochromosomes to the CNM-41 sequences characteristic of microchromosomes (Fig. 2f–h); additionally, we identified centromeres from Chr5, ChrZ, and Chr27 in the Silkie genome that lack tandem repeat sequences (Fig. 1a and b), which aligns with results from previous studies [62, 71]. Our examination of repetitive sequences revealed that the heterochromatic W chromosome serves as a refuge for ERVs (Fig. 3c), which is consistent with prior research [72]. The predominant types of ERVs differ between chickens and ducks: ERV1 is most prevalent on Silkie ChrW, whereas Mallard ChrW is more highly represented by both ERV1 and ERVL (Fig. 3d). We conducted a quantitative analysis of 5mC methylation levels across the genome and discovered that telomeric and centromeric regions, gene-poor areas rich in repetitive sequences, exhibit significantly greater methylation than does the overall genome (Fig. 4c). These regions, known as constitutive heterochromatin, exhibit relatively high levels of methylation, as revealed by a study involving 13 various bird species from 10 families across 7 orders [73]. Our results also revealed that sex chromosomes and microchromosomes present elevated levels of 5mC methylation. Another study [74] also indicated that the W chromosome and dense chromosomes in chicken genomes present increased 5mC methylation. Interestingly, only the 5' UTR regions of genes presented significantly lower methylation (Fig. 4d). This region contains various regulatory elements, which are mostly associated with the promoter region [64], indicating their involvement in regulating gene expression through 5mC methylation. This study quantified 5mC methylation levels only in DNA exclusively from blood; further investigation across additional tissues and developmental stages may be necessary for comprehensive validation.

Ultimately, we recovered 401 (70.20%) missing genes from this study and 325 (56.92%) missing genes from Silkie and Mallard genomes, including the first identification of *TNFA* in ducks, revealing diverse expression trends across tissues. Compared with CAU_Silkie_1.0 and CAU_Wild_1.0, our current assemblies, CAU_Silkie_2.0 and CAU_Wild_2.0, significantly increased the number of identified missing genes, with 165 (150%) and 203 (271%) more missing genes, respectively.

Birds represent over 30% of known tetrapod diversity [75], and the chicken (*Gallus gallus*) and duck (*Anas platyrhynchos*) are two important model species for scientific discovery in developmental biology, genetics, virology, and immunology [76–78]. Two near-complete avian genomes provide important data for avians to solve important biological problems in some fields such as missing genes, avian genome evolution, and avian phenotypic diversity. Chicken and duck are the two most widely studied poultry species, but some genes with important functions have not been previously annotated, such as *TNFA* in ducks and other genes annotated in this paper for the first time (Table S19), and the near T2T genomes and annotations of Silkie and Mallard will lay a valuable database for the functional and evolutionary analyses of these annotated genes and their related economic traits.

## Conclusion

In conclusion, the successful near T2T assemblies of the Mallard and Silkie, including the novel reconstruction of gap-free sex chromosomes in ducks, have profoundly enriched our comprehension of avian genetic architecture. This study reveals the differences among various chromosome types concerning centromeres, repetitive sequences, and methylation patterns. Moreover, the identification and annotation of previously thought-to-be missing genes lay the groundwork for future research aimed at exploring their functional significance. This work not only demonstrates the importance of T2T genomes but also provides a theoretical foundation for investigating the functions of missing genes.

## Supplementary Information

Additional file 1: Fig. S1. Summary of sequencing data from CAU_Wild_2.0 and CAU_Silkie_2.0. Fig. S2. Genome assembly pipeline of CAU_Silkie_2.0. Fig. S3. Genome assembly pipeline of CAU_Wild_2.0. Fig. S4. The collinearity between chromosome W of CAU_Silkie_2.0 and scaffolds from CAU_Silkie_1.0. Fig. S5. Hi-C interaction signal heatmap of CAU_Silkie_2.0. Fig. S6. Average GC content of chromosomes and new sequences of a. CAU_Wild_2.0 and b. CAU_Silkie_2.0 compared with the previous assembly version. Fig. S7. Hi-C interaction signal heatmap of CAU_Wild_2.0. Fig. S8. Locations of centromeres,  telomeres, and gaps on chromosomes of CAU_Wild_1.0. Fig. S9. Locations of centromeres, telomeres, and gaps on chromosomes of SKLA1.0. Fig. S10. The gene collinearity of sex chromosomes between CAU_Wild_2.0 and CAU_Wild_1.0 and comparison of annotated genes. Fig. S11. Similarity heatmap of the centromere of Chr5 of CAU_Wild_2.0. Fig. S12. Locations of "missing" genes found on chromosomes of CAU_Silkie_2.0. Fig. S13. Locations of "missing" genes found on chromosomes of CAU_Wild_2.0. Fig. S14. The expression levels of *TNFA* in 19 different duck tissues.

Additional file 2: Table S1. An overview of the sequencing data for Mallard genomes. Table S2. An overview of the sequencing data for Silkie genomes. Table S3. RNA-seq data was utilized for gene annotation in the Silkie and Mallard genomes. Table S4. Annotation of non-coding RNA within the Silkie and Mallard genomes. Table S5. Annotation of repetitive sequences within the Silkie and Mallard genomes. Table S6. A comparison of contigs continuity during the assembly process of the Mallard genome. Table S7. A comparison of contigs continuity during the assembly process of the Silkie genome. Table S8. A continuity comparative analysis of chicken and duck genomes. Table S9. A description of the chromosomal status of the Silkie genome. Table S10. The length of newly assembled sequences relative to the version 1.0 genome across each chromosome of Silkie and Mallard genomes. Table S11. A description of the chromosomal status of the Mallard genome. Table S12. The location of centromeric regions in the Mallard genome. A description of the chromosomal status of the Silkie genome. Table S13. Distribution of centromeric regions in Silkie genome identified via CENPA ChIP-seq. Table S14. The BUSCO scores of newly assembled genomes and reference genomes. Table S15. Genes annotated in the Silkie and Mallard genomes across various databases. Table S16. BUSCO scores of annotation from Silkie and Mallard genomes. Table S17. Gene annotation of the ZW chromosomes in the Mallard. Table S18. Genes annotated to ChrZ and ChrW from CAU_Wild_2.0 and CAU_Wild_1.0. Table S19. Review of the 'missing genes' with Silkie and the Mallard genomes. Table S20. The RNA-seqs are used in the quantification of gene expression.

## Data Availability

The final genome assembly and raw sequencing reads were deposited at NCBI under BioProject PRJNA799866 and PRJNA554956. The RNA-seq datasets used in this study are reported in Tables S3 and S19. The annotation information and the code used in this study were deposited in the Zenodo database. (https://doi.org/10.5281/zenodo.12721248).

## Abbreviations

BOMs: BioNano Optical Maps
BUSCO: Benchmarking Universal Single-Copy Orthologs
CCS: Circular consensus sequencing
CENPA: Centromere protein A
CLR: Continuous Long read
CR1: Chicken repeat 1
ERVs: Endogenous retroviruses
ERVL: Endogenous retroviruses L
EVM: EVidenceModeler
ERV1: Endogenous retroviruses 1
HiFi: High-fidelity
Hi-C: High-throughput chromatin conformation capture
kb: Kilobase
LINEs: Long interspersed nuclear elements
Mb: Megabase
miRNA: MicroRNA
ONT: Oxford Nanopore Technology
ORFs: Open reading frames
PacBio: Pacific Biosciences
QV: Quality value
rRNA: Ribosomal RNA
snRNA: Small nuclear RNA
tRNA: Transfer RNA
T2T: Telomere-to-telomere
TNFA: Tumor necrosis factor alpha
5mC: 5-Methylcytosine
5′ UTR: 5′ Untranslated region
