# Supplementary material for: Near telomere-to-telomere genome assemblies of Silkie Gallus gallus and Mallard Anas platyrhynchos restored the structure of chromosomes and “missing” genes in birds
Source: J Anim Sci Biotechnol. 2025 Jan 20;16:9. doi: 10.1186/s40104-024-01141-1 (PMC11745021; doi:10.1186/s40104-024-01141-1)
Supplement: Supplementary file 1 — Additional file 1: Fig. S1. Summary of sequencing data from CAU_Wild_2.0 and CAU_Silkie_2.0. Fig. S2. Genome assembly pipeline of CAU_Silkie_2.0. Fig. S3. Genome assembly pipeline of CAU_Wild_2.0. Fig. S4. The collinearity between chromosome W of CAU_Silkie_2.0 and scaffolds from CAU_Silkie_1.0. Fig. S5. Hi-C interaction signal heatmap of CAU_Silkie_2.0. Fig. S6. Average GC content of chromosomes and new sequences of a. CAU_Wild_2.0 and b. CAU_Silkie_2.0 compared with the previous assembly version. Fig. S7. Hi-C interaction signal heatmap of CAU_Wild_2.0. Fig. S8. Locations of centromeres, telomeres, and gaps on chromosomes of CAU_Wild_1.0. Fig. S9. Locations of centromeres, telomeres, and gaps on chromosomes of SKLA1.0. Fig. S10. The gene collinearity of sex chromosomes between CAU_Wild_2.0 and CAU_Wild_1.0 and comparison of annotated genes. Fig. S11. Similarity heatmap of the centromere of Chr5 of CAU_Wild_2.0. Fig. S12. Locations of "missing" genes found on chromosomes of CAU_Silkie_2.0. Fig. S13. Locations of "missing" genes found on chromosomes of CAU_Wild_2.0. Fig. S14. The expression levels of TNFA in 19 different duck tissues. [file 40104_2024_1141_MOESM1_ESM.pdf]

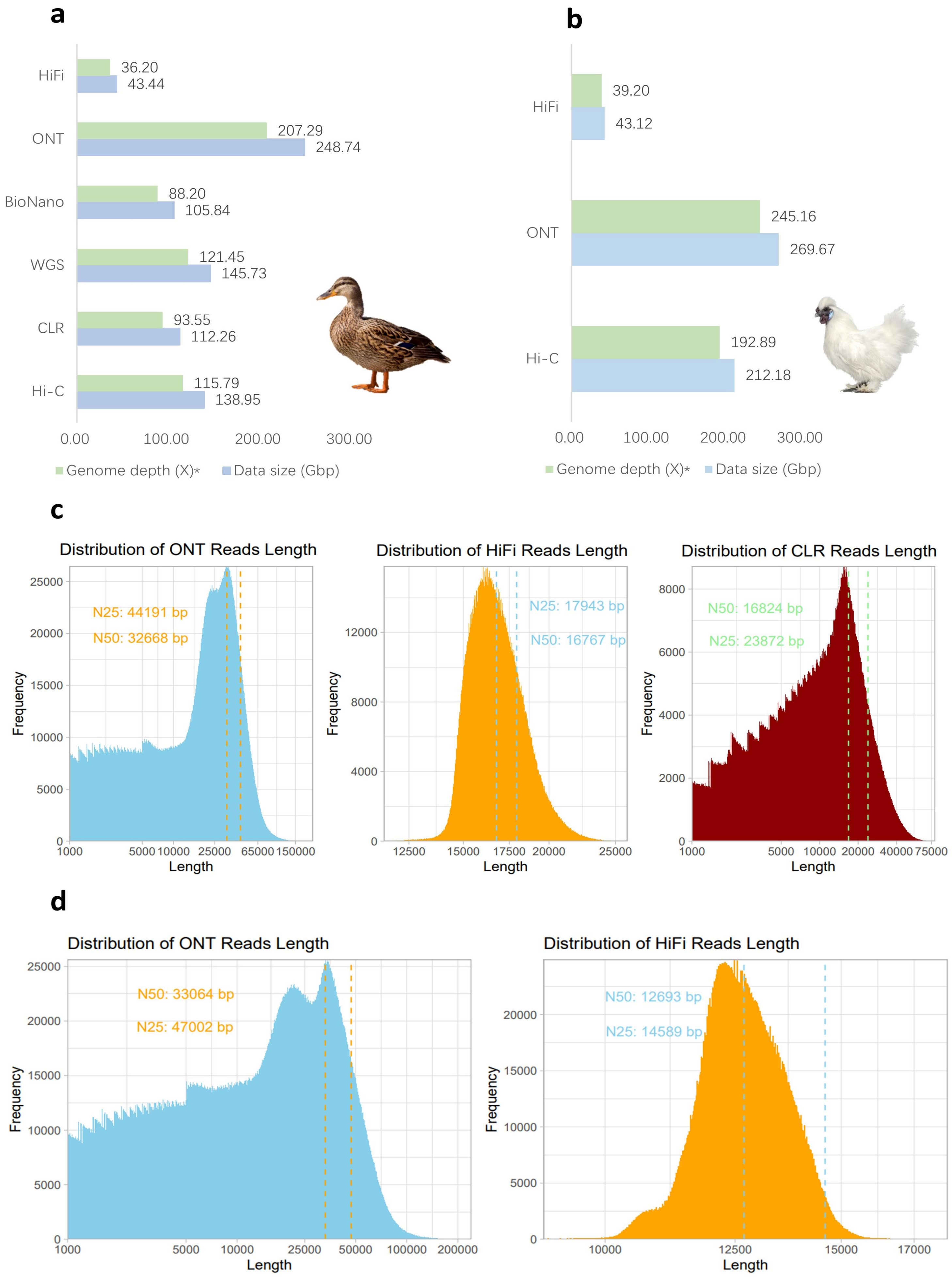

Fig. S1. Summary of sequencing data from CAU_Wild_2.0 and CAU_Silkie_2.0. **a.** Genome depth and data size of Mallard sequencing reads; **b.** genome depth and data size of Silkie sequencing reads; **c.** distribution of reads length for Mallard; **d.** distribution of reads length for Silkie.

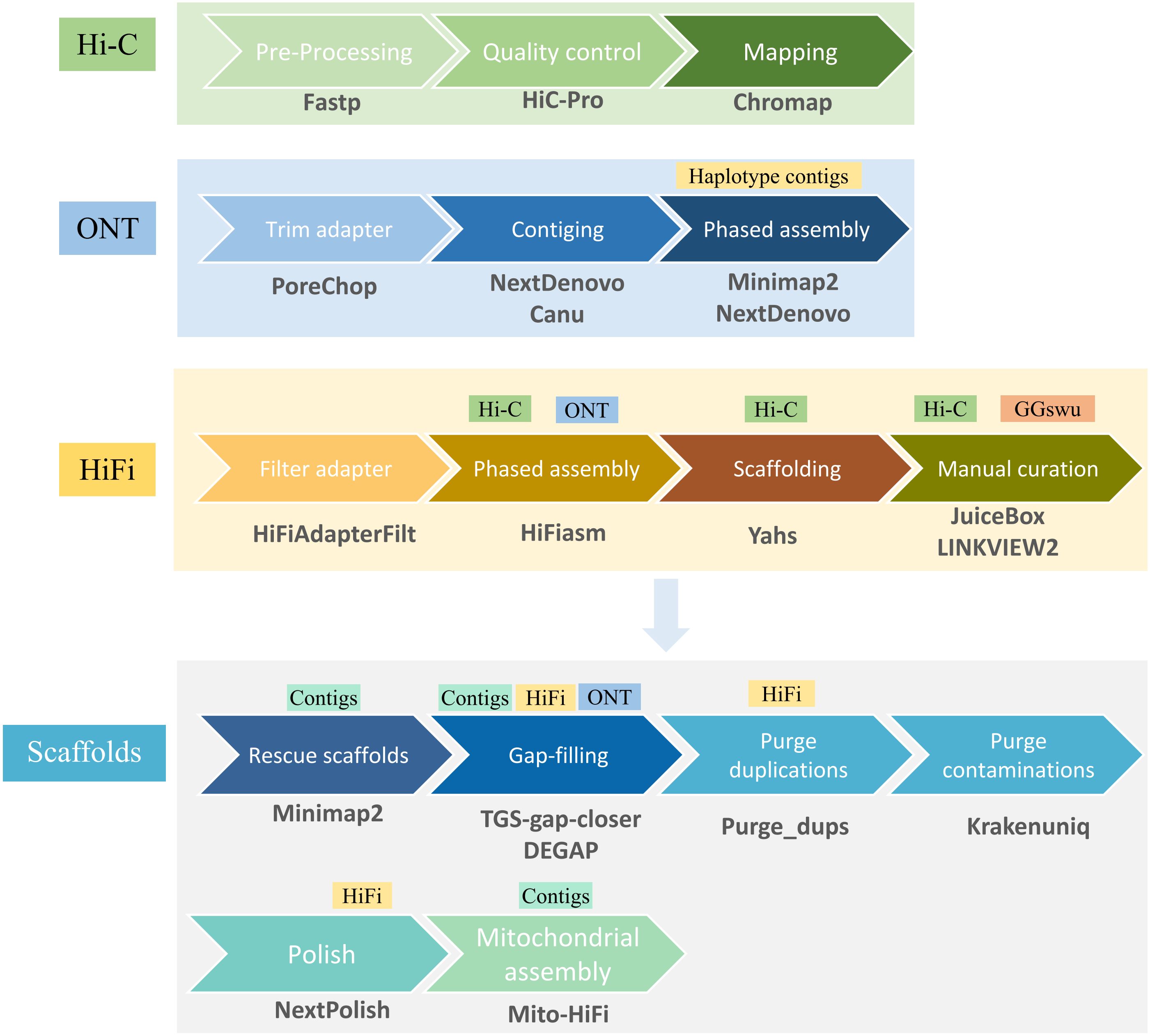

Fig. S2. Genome assembly pipeline of CAU_Silkie_2.0. The gray text beneath the polygon indicates the software utilized in that particular process. In contrast, the text above the polygon signifies the input data employed in that specific process.

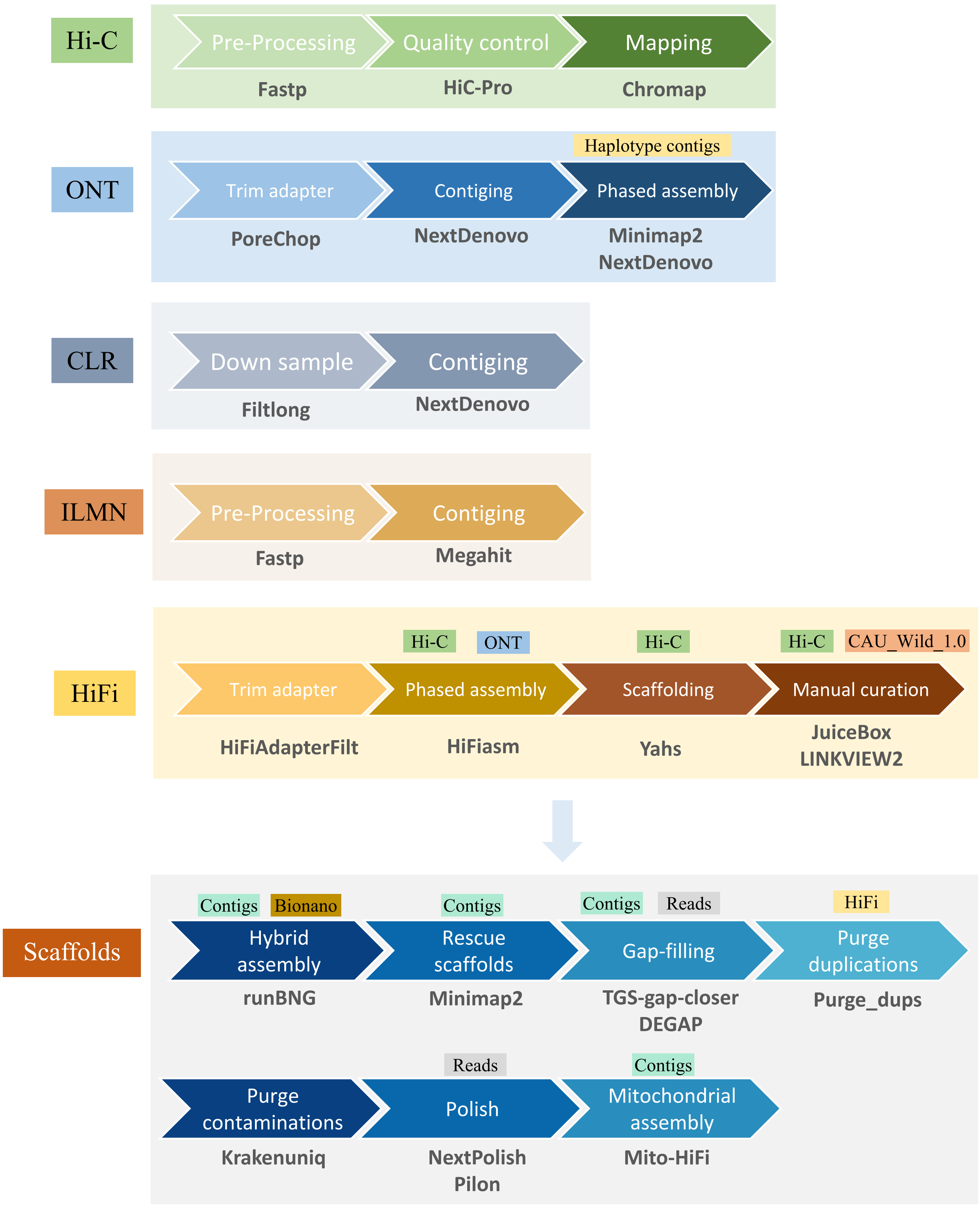

Fig. S3. Genome assembly pipeline of CAU_Wild_2.0. The gray text beneath the polygon indicates the software utilized in that particular process. In contrast, the text above the polygon signifies the input data employed in that specific process.

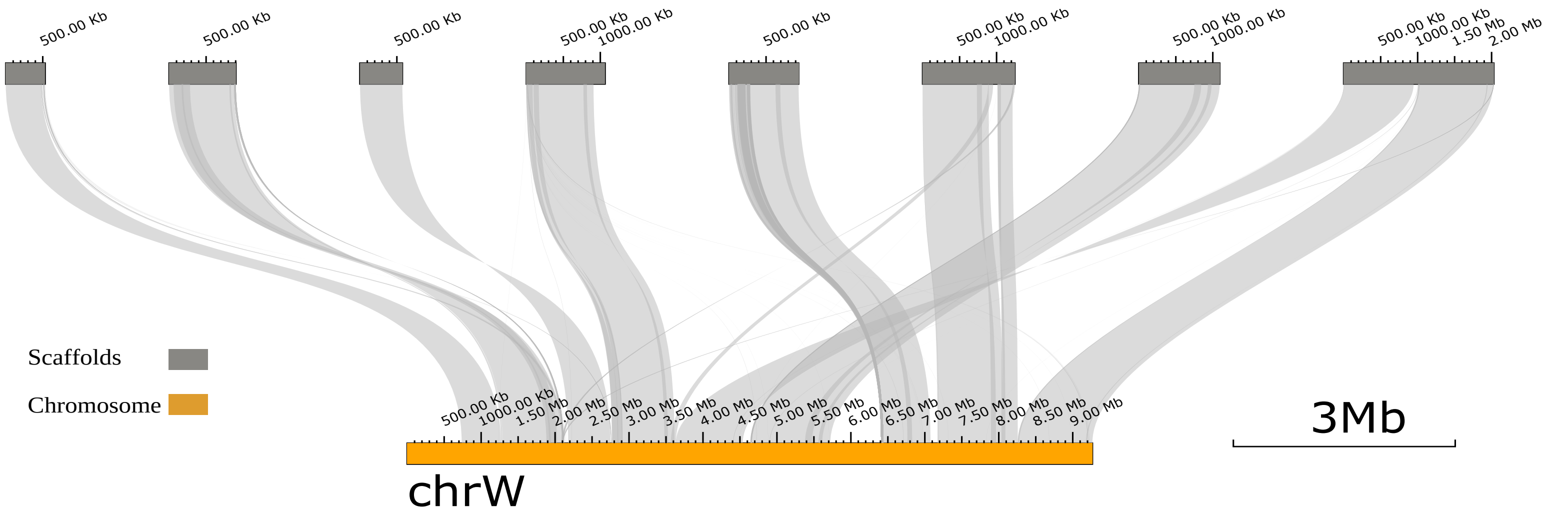

Fig. S4. The collinearity between chromosome W of CAU_Silkie_2.0 and scaffolds from CAU_Silkie_1.0.

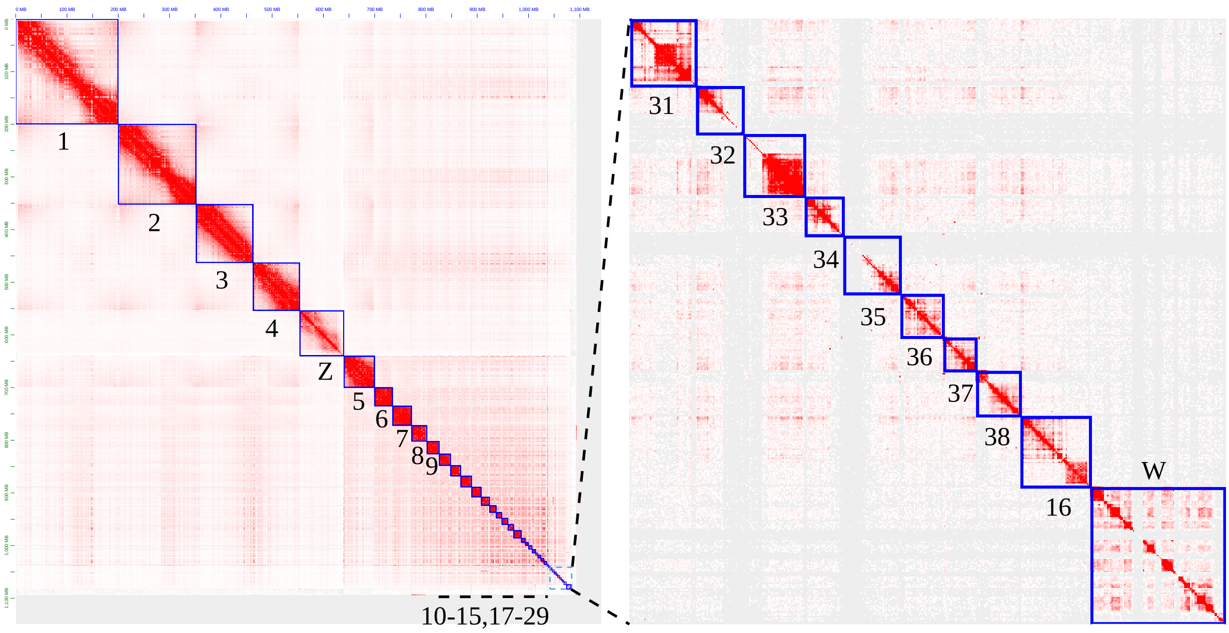

Fig. S5. Hi-C interaction signal heatmap of CAU_Silkie_2.0.

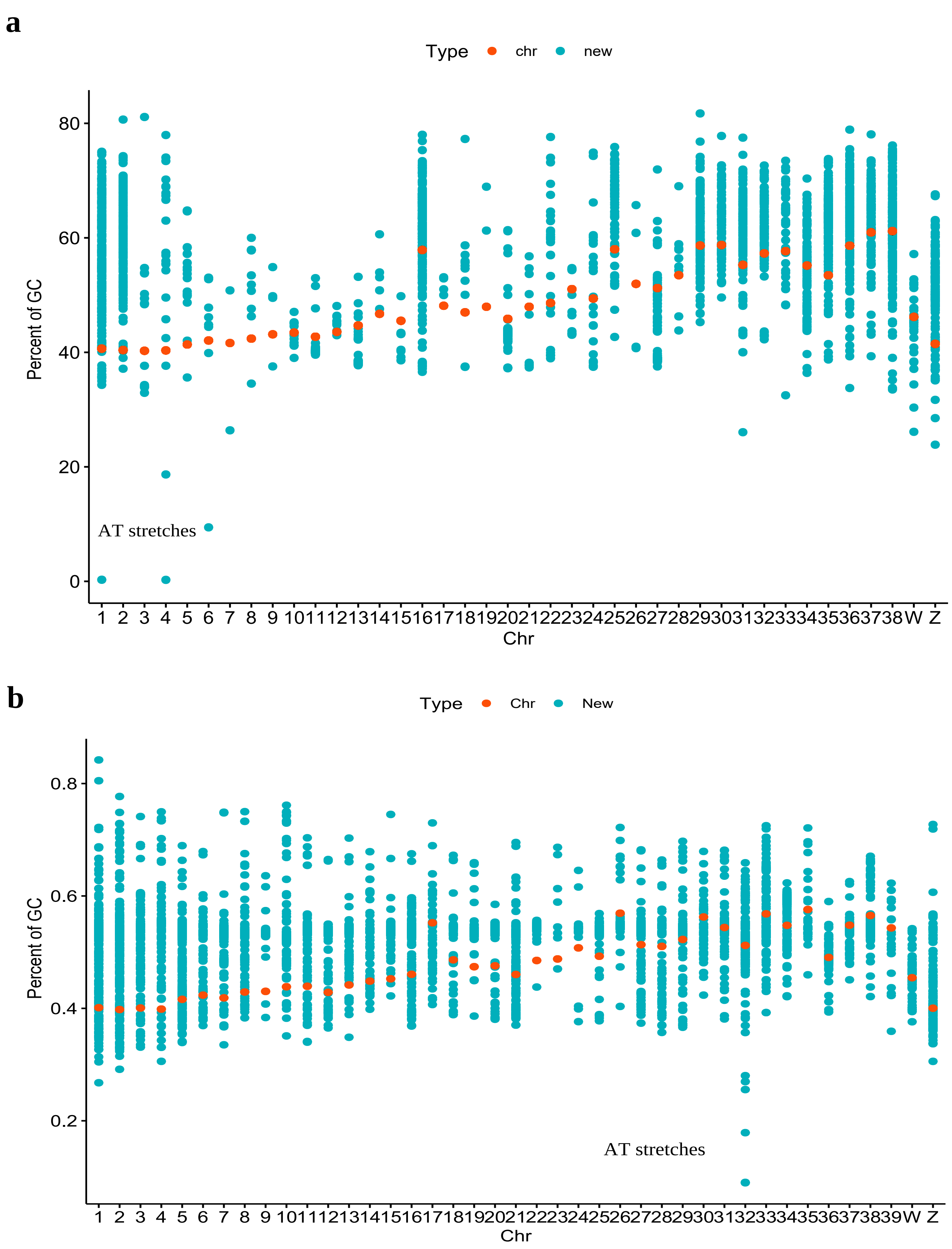

Fig. S6. Average GC content of chromosomes and new sequences of **a.** CAU_Wild_2.0 and **b.** CAU_Silkie_2.0 comparaed with previous assembly version. Red dot indicates average GC content of chromosomes.

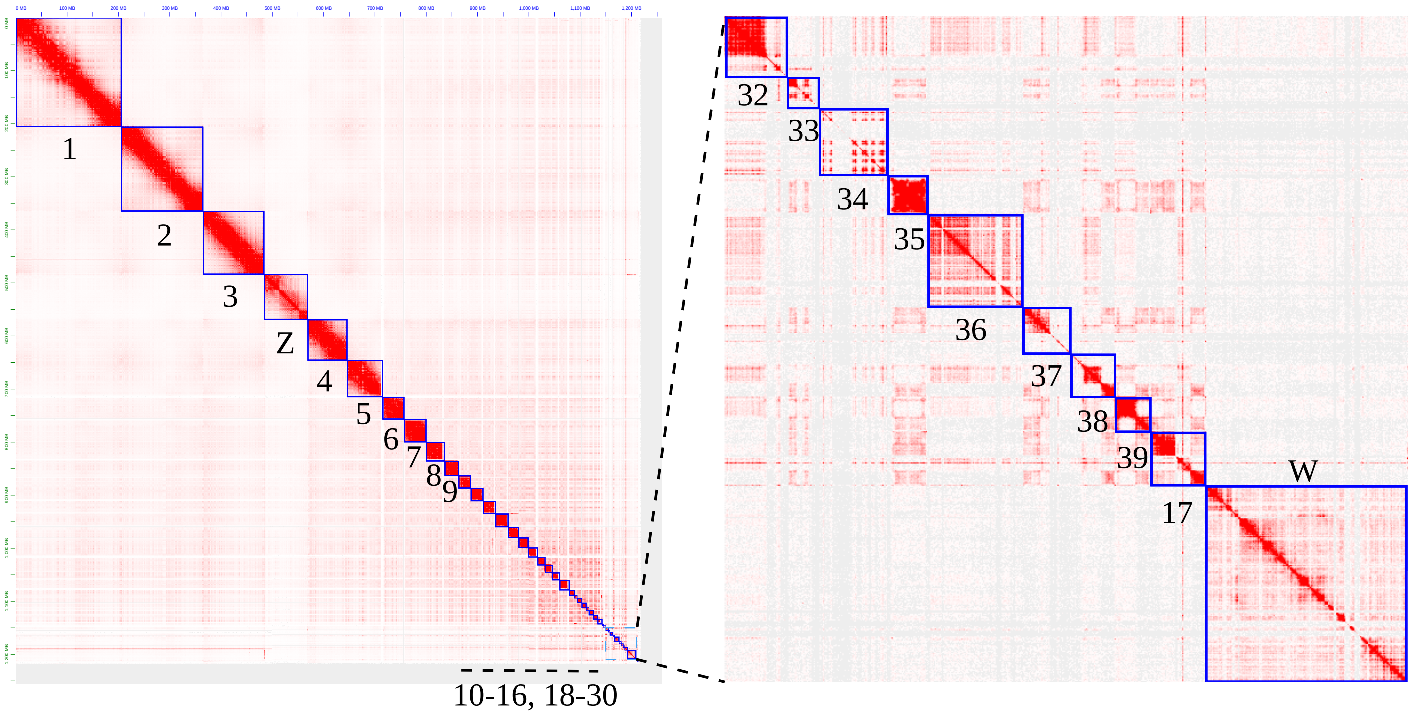

Fig. S7. Hi-C interaction signal heatmap of CAU_Wild_2.0.

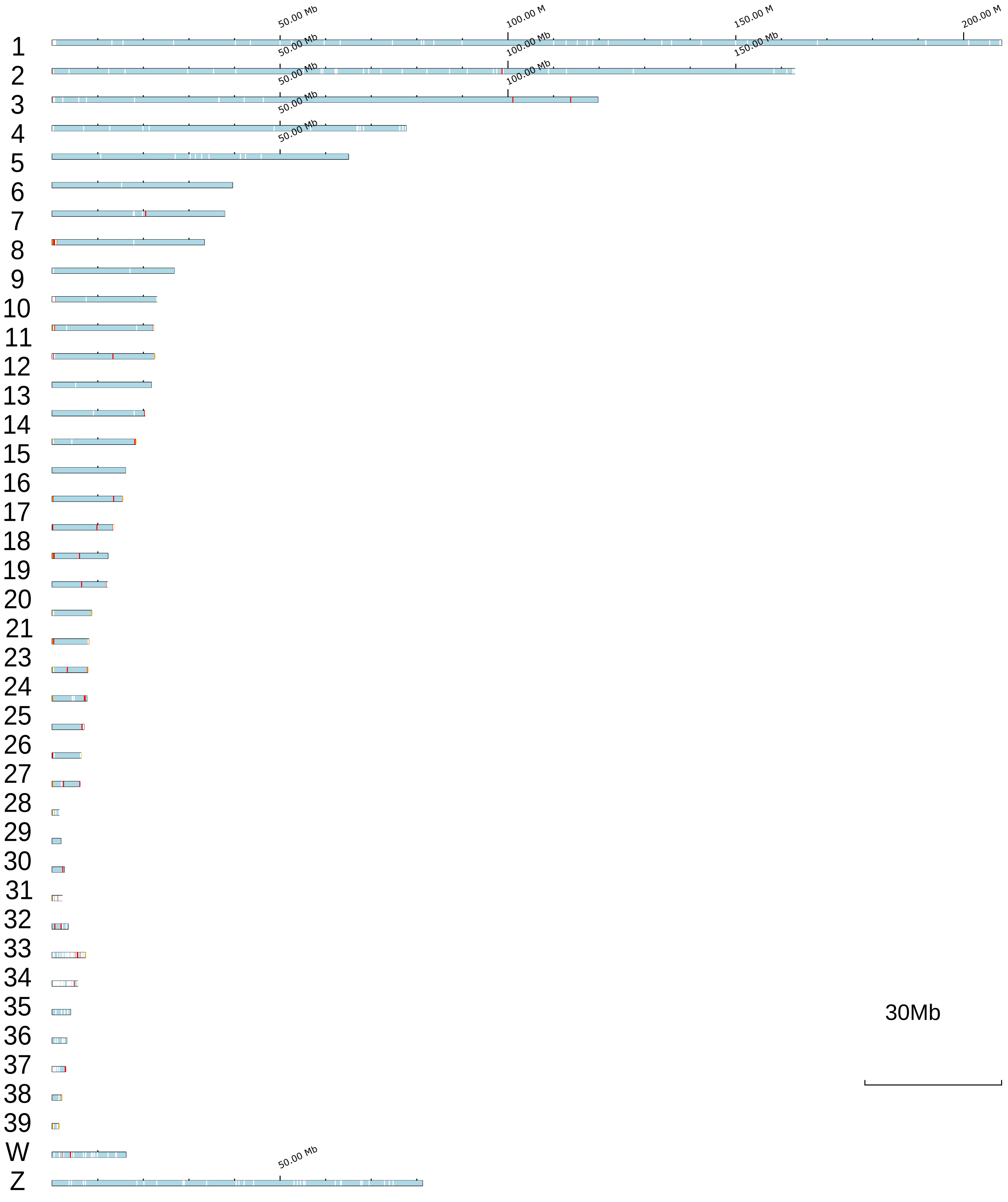

Fig. S8. Locations of centromeres, telomeres, and gaps on chromosomes of CAU_Wild_1.0. Centromeric squences are ploted by red color, telomeric sequences are ploted by orange color and gaps are ploted by white speces.

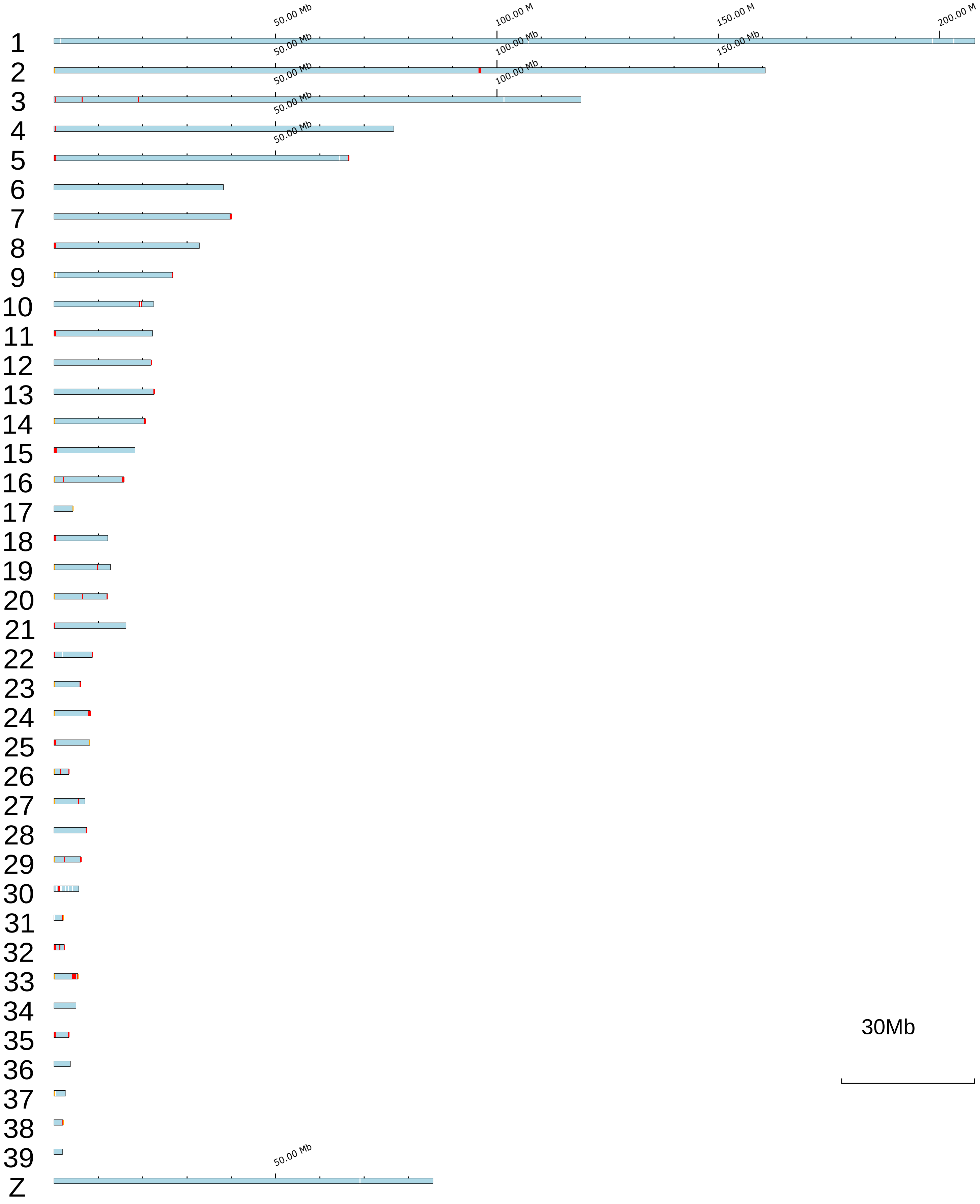

Fig. S9. Locations of centromeres, telomeres, and gaps on chromosomes of SKLA1.0. Centromeric squences are ploted by red color, telomeric sequences are ploted by orange color and gaps are ploted by white speces.

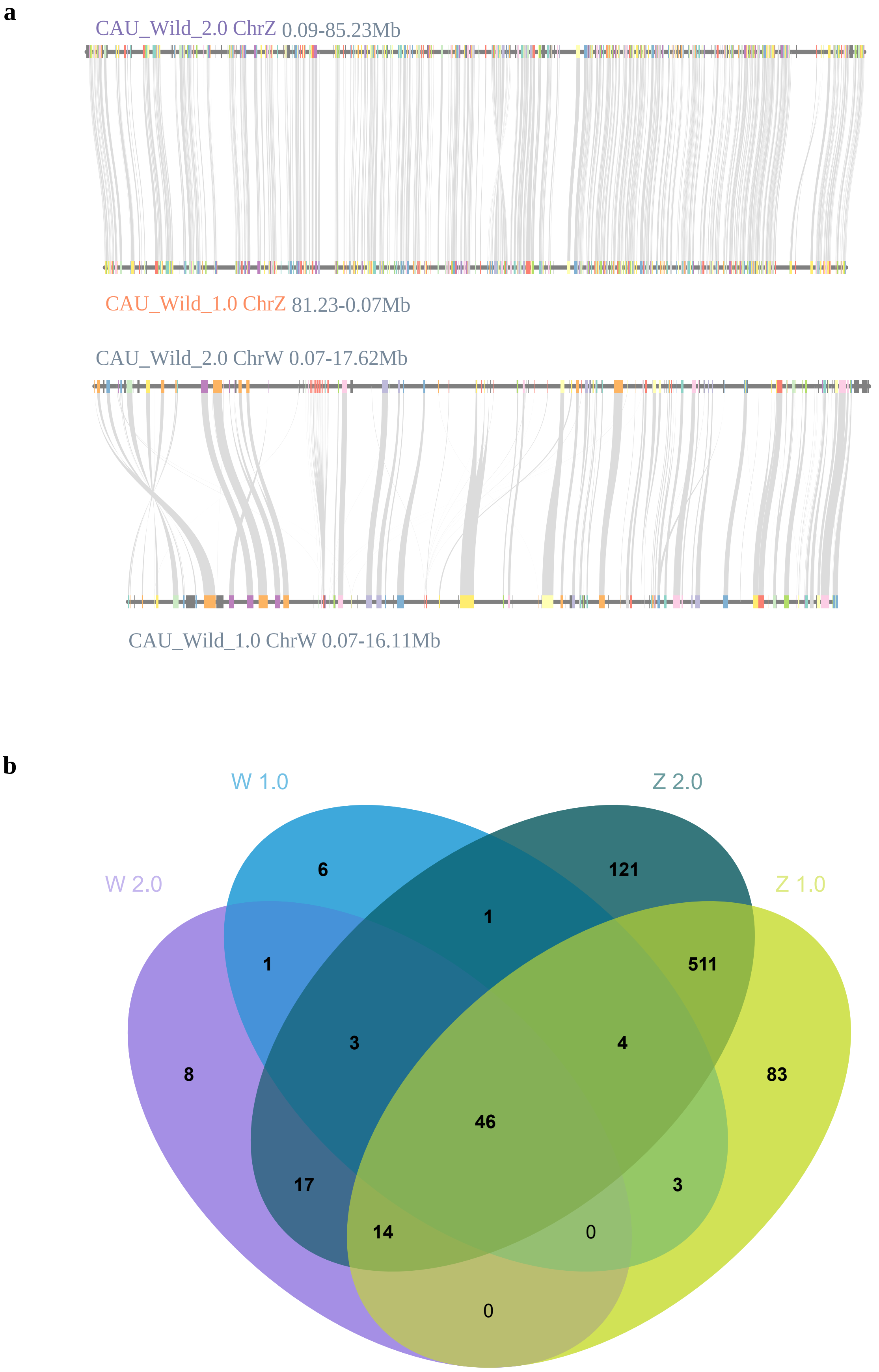

Fig. S10. The gene collinearity of sex chromosome between CAU_Wild_2.0 and CAU_Wild_1.0 and comparison of annotated genes. **a.**The collinearity of sex chromosome genes between CAU_Wild_2.0 and CAU_Wild_1.0. Genes with collinearity that have the same color are orthologous genes. **b.**The annotated genes of sex chromosome between CAU_Wild_2.0 and CAU_Wild_1.0.

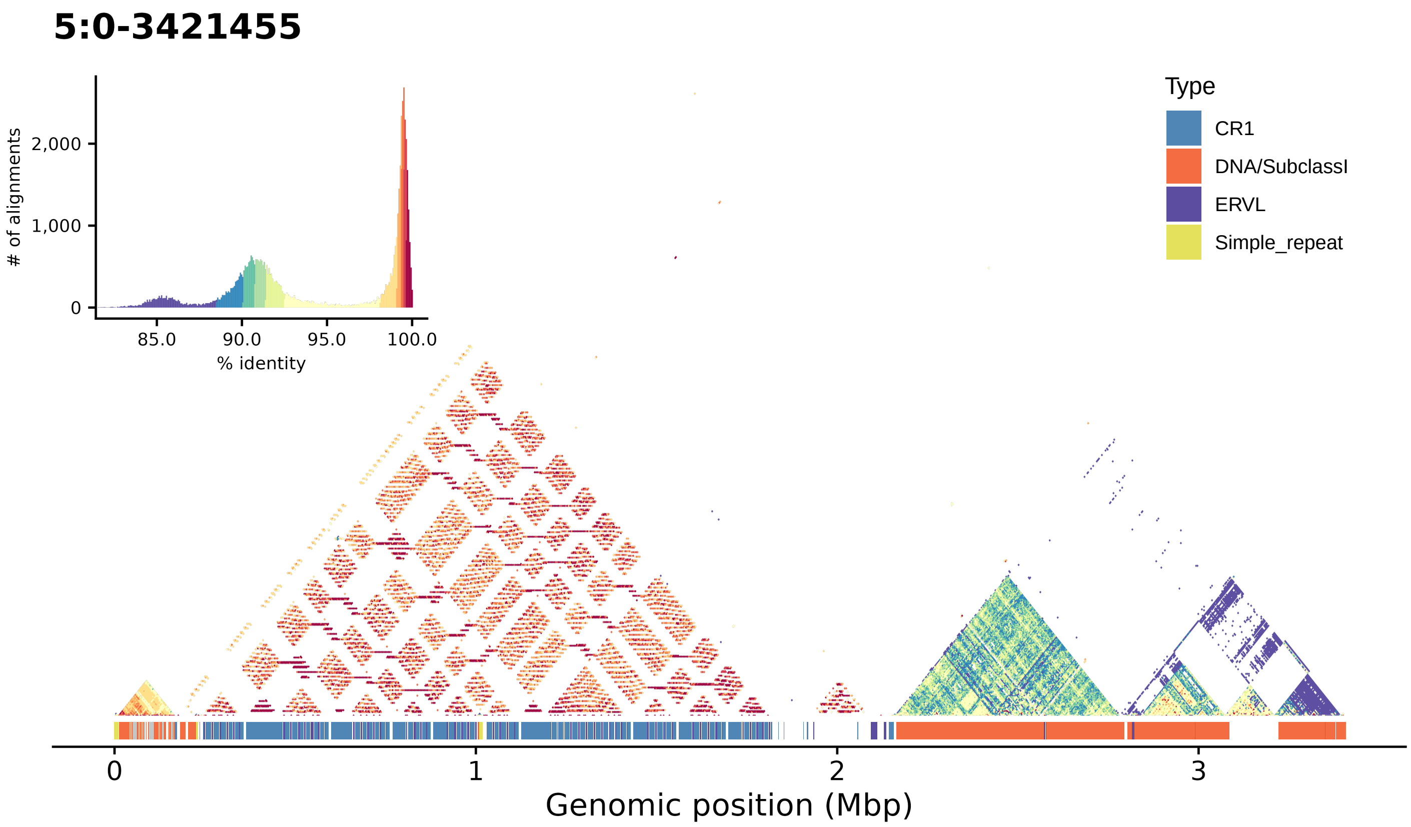

Fig. S11. Similarity heatmap of centromere of Chr5 of CAU_Wild_2.0. Main repeat types are noted at Legend.

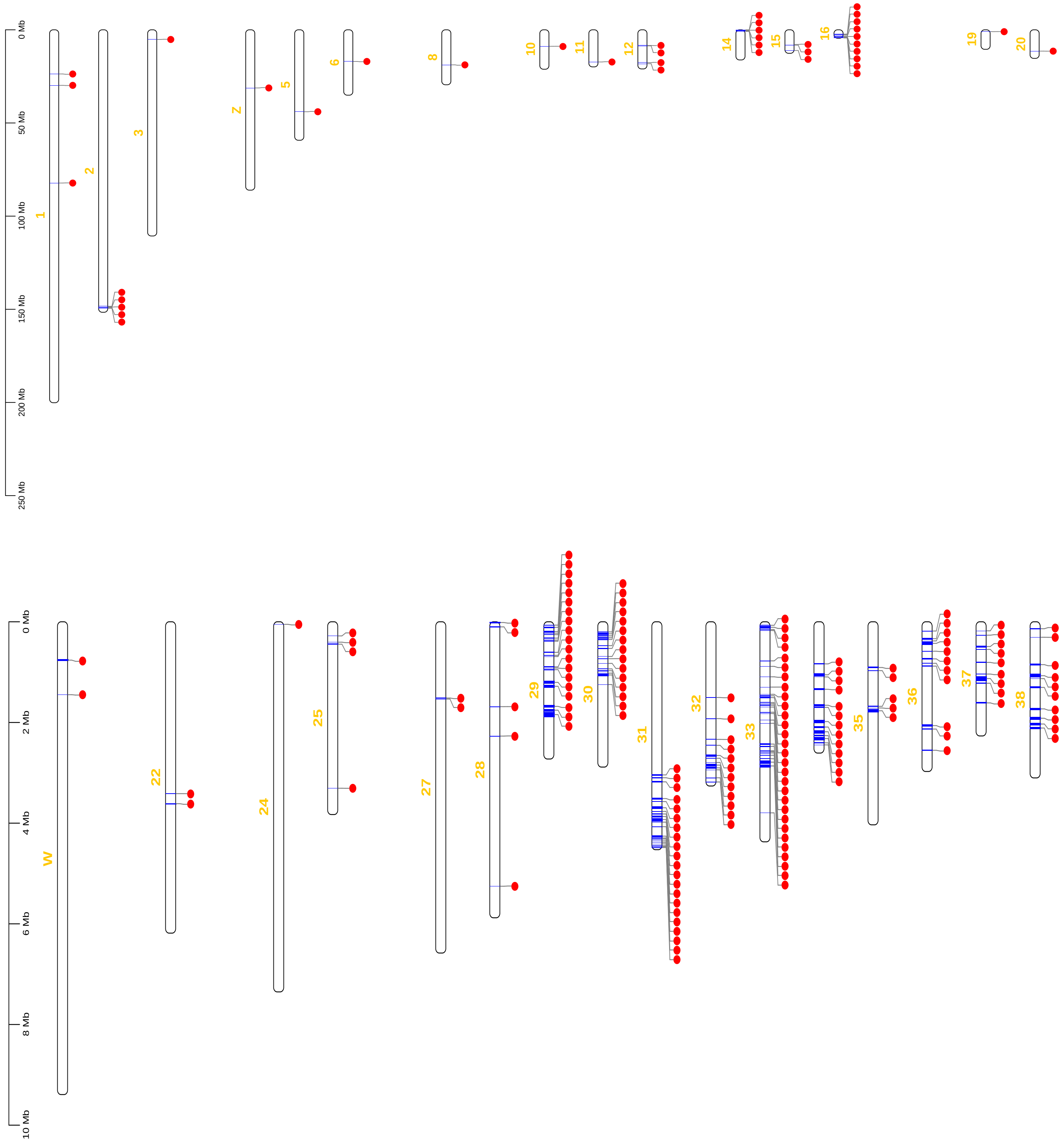

Fig. S12. Locations of "missing" genes founded on chromosomes of CAU_Silkie_2.0. Red dots represent "missing" genes founded on chromosomes, the blue band indicates location.

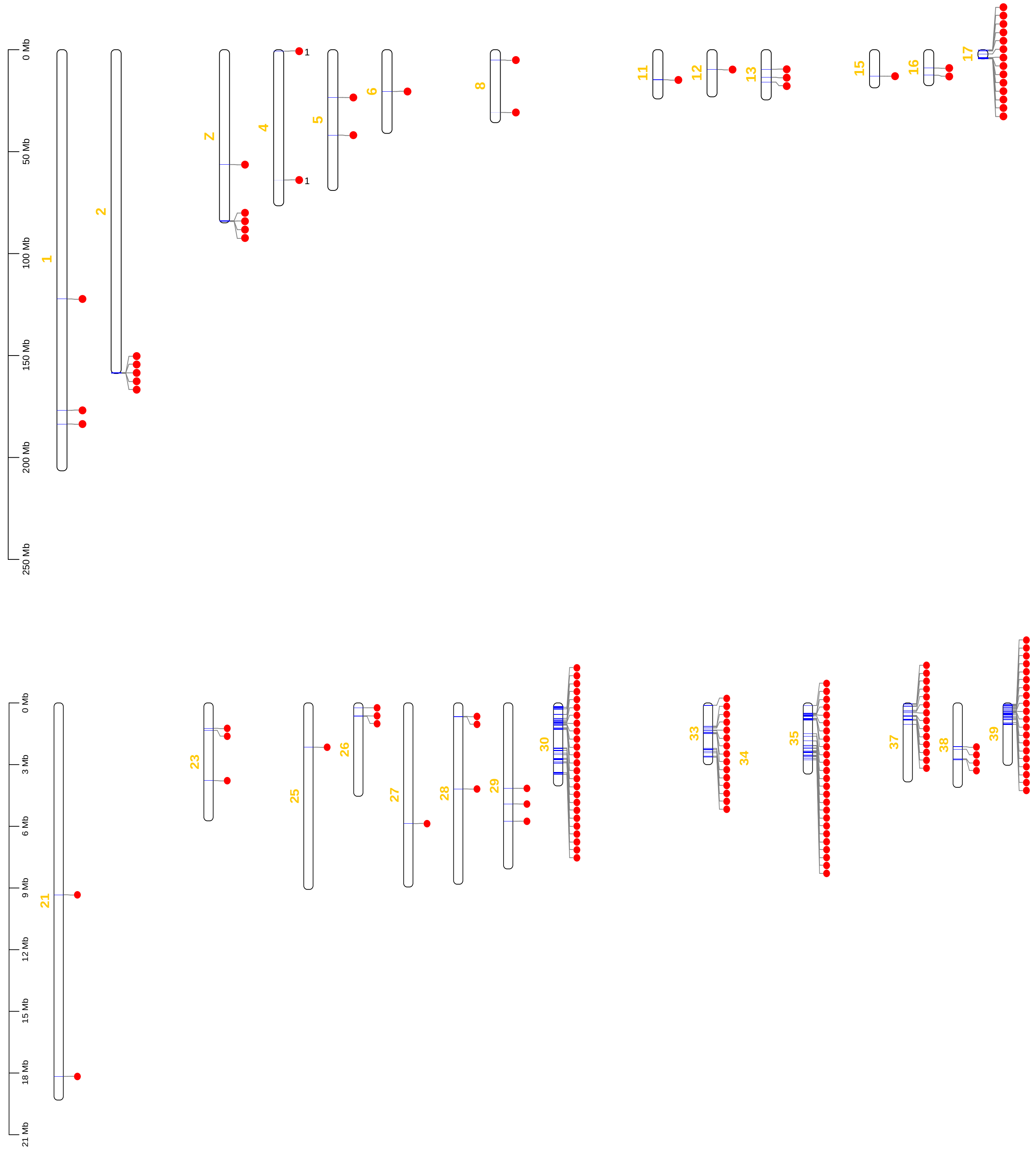

Fig. S13. Locations of "missing" genes founded on chromosomes of CAU_Wild_2.0. Red dots represent "missing" genes founded on chromosomes, the blue band indicates location.

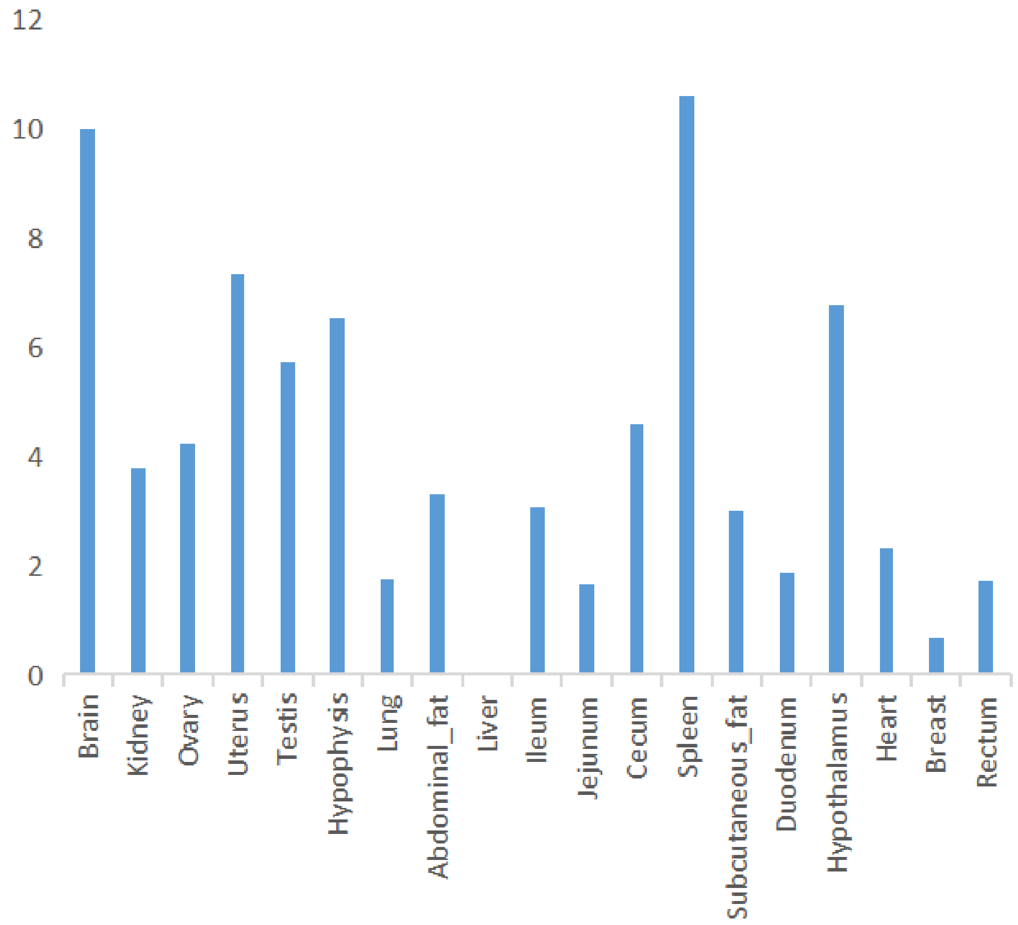

Fig. S14. The expression levels of *TNFA* in 19 different duck tissues. In units of TPM (Transcripts Per Million).
